# Can strenuous exercise harm the heart? Insights from a study of cardiovascular neural regulation in amateur triathletes

**DOI:** 10.1371/journal.pone.0216567

**Published:** 2019-05-07

**Authors:** Laura Adelaide Dalla Vecchia, Franca Barbic, Beatrice De Maria, Domenico Cozzolino, Roberto Gatti, Franca Dipaola, Enrico Brunetta, Antonio Roberto Zamuner, Alberto Porta, Raffaello Furlan

**Affiliations:** 1 IRCCS Istituti Clinici Scientifici Maugeri, Milan, Italy; 2 Humanitas Clinical and Research Center, Humanitas University, Rozzano, Milan, Italy; 3 Department of Internal Medicine, University of Campania "L. Vanvitelli”, Napoli, Italy; 4 Departmento de Kinesiología, Universidad Católica del Maule, Talca, Chile; 5 Department of Cardiothoracic, Vascular Anesthesia and Intensive Care, IRCCS Policlinico San Donato, San Donato Milanese, Milan, Italy; 6 Department of Biomedical Sciences for Health, University of Milan, Milan, Italy; Universidade Federal de Juiz de Fora, BRAZIL

## Abstract

Regular exercise is recommended to improve the cardiovascular risk profile. However, there is growing evidence that extreme volumes and intensity of long-term exertion may increase the risk of acute cardiac events. The aim of this study is to investigate the after-effects of regular, strenuous physical training on the cardiovascular neural regulation in a group of amateur triathletes compared to age-matched sedentary controls. We enrolled 11 non-elite triathletes (4 women, age 24±4 years), who had refrained from exercise for 72 hours, and 11 age-matched healthy non-athletes (3 women, age 25±2 years). Comprehensive echocardiographic and cardiopulmonary exercise tests were performed at baseline. Electrocardiogram, non-invasive blood pressure, respiratory activity, and muscle sympathetic nerve activity (MSNA) were continuously recorded in a supine position (REST) and during an incremental 15° step-wise head-up tilt test up to 75° (TILT). Blood samples were collected for determination of stress mediators. Autoregressive spectral analysis provided the indices of the cardiac sympathetic (LF_RR_) and vagal (HF_RR_) activity, the vascular sympathetic control (LF_SAP_), and the cardiac sympatho-vagal modulation (LF/HF). Compared to controls, triathletes were characterized by greater LF_RR_, LF/HF ratio, LF_SAP_, MSNA, and lower HF_RR_ at REST and during TILT, i.e. greater overall cardiovascular sympathetic modulation together with lower cardiac vagal activity. Cortisol and adrenocorticotropic hormone concentrations were also higher in triathletes. In conclusion, triathletes were characterized by signs of sustained cardiovascular sympathetic overactivity. This might represent a risk factor for future cardiovascular events, given the known association between chronic excessive sympathetic activity and increased cardiovascular risk.

## Introduction

Regular physical activity is a positive determinant of longevity and is associated with an improved cardiovascular risk [1]. However, prescription of exercise may be complex. The Paracelsus concept of dose-related toxic effects of drugs has been recently extended to sports [2, 3], advocating the need to establish the ‘dose’ of exercise that might be even harmful, rather than protective. Vigorous exertion may in-turn transiently increase the risk of acute cardiac events in elite and non-elite athletes [2–6], of atrial fibrillation for endurance sports’ practitioners [7], as well as of transient cardiovascular modifications and permanent remodeling in ultra-marathon [8], marathon [9], and half-marathon [10] runners. The level of exercise to be recommended at different ages, in the presence of cardiovascular risk, and/or diagnosed cardiovascular disease is also under discussion [1, 11]. Exercise intensity may play a critical role as a potential noxious factor not only during races, but also during training sessions. In this perspective, the understanding of exercise-induced modifications in the neural mechanisms controlling the cardiovascular system might play an important role in tailoring the exercise dose for each individual. A shift towards a predominance of sympathetic modulation to the heart and vessels has been reported in endurance exercise [10, 12–14]. On the other hand, these modifications together with a decreased cardiac baroreflex sensitivity are independent risk factors for increased cardiac mortality after a myocardial infarction [15, 16]. It is therefore critical to assess whether or not athletes may exhibit a habitual exceeding cardiovascular sympathetic tone resulting from bouts of strenuous exercise that might theoretically increase their overall cardiovascular risk profile.

The aim of the present study was to test this hypothesis comparing a group of adult non-elite triathletes, far away from the last bout of physical exercise, to healthy age-matched sedentary controls. Triathlon is an increasingly popular and practiced outdoor endurance sport. Participants must complete three different and consecutive tasks, i.e. swimming, cycling and running. This is accomplished by intense training sessions 5 to 6 days per week with a short recovery time possibly inducing a sustained neural sympathetic overactivity. We investigated the changes in the cardiovascular autonomic profile by means of the analysis of i) heart rate (HR) and systolic arterial pressure (SAP) variability, ii) muscle sympathetic nerve activity (MSNA), iii) circulating levels of stress mediators, i.e. neurotransmitters, peptides, and steroid hormones, in supine resting conditions and during passive orthostatism. Analyses of MSNA, and HR and SAP variability have been demonstrated capable of detecting the complex adaptive sympathovagal balance adjustments to various challenges induced by exercise and recovery in athletes [12, 13].

## Methods

The study enrolled 11 amateur triathletes and 11 age-matched healthy non-athletes. All subjects were healthy volunteers without any family history of cardiovascular disease or sudden death. None of them was taking any prescribed medication, nor reported the use of doping substances using an anonymous questionnaire. All subjects were carefully interviewed to exclude the presence of signs of overreaching (OR) or overtraining (OT) [17].

All participants underwent a comprehensive echocardiographic examination performed in accordance with the American Society of Echocardiography recommendations [18] to exclude any structural heart disease, and a cardiopulmonary exercise test (CPET) in accordance with the American Heart Association Guide [19] to evaluate their physical conditioning. The protocol was conducted during the competitive race season to ensure a high fitness level. The triathletes had been competing for at least 2 years in triathlon and were training five to six times per week. They were studied 72 hours far away from the last bout of physical exercise to avoid the short-term autonomic and cardiovascular confounding after-effects induced by recent training sessions [13]. In addition, none of them had participated to a competition for at least 2 weeks. Control subjects were sedentary individuals who did not perform any regular physical activity. The study conformed to the standards set by the declaration of Helsinki and ethical approval was obtained from Sacco Hospital Ethics Committee. All subjects provided written, informed consent.

### Experimental protocol

All subjects were tested in a quiet room in the morning. A light, caffeine-free, breakfast was consumed at least 2 hours before the test, after a good night's sleep.

Electrocardiogram (ECG) and non-invasive blood pressure (BP) (Finapres, Finapres Medical System) signals were continuously recorded. The subject's arm was fixed to the thorax to maintain the hand at the level of the heart during the upright position. Intermittent brachial BP was measured with a manual sphygmomanometer on the contralateral arm. Respiratory activity was concomitantly evaluated by a thoracic bellow connected to a pressure transducer. MSNA was recorded from the peroneal nerve of the right leg, as detailed elsewhere [20]. The raw nerve signal was band-pass filtered (700–2000 Hz), amplified (100 × 999.9), rectified, and integrated (time constant of 0.1 s) to obtain mean voltage MSNA using a nerve traffic analysis system (Nerve Traffic Analyzer; University of Iowa Bioengineering, Iowa City, IA).

ECG, BP, respiratory activity, and integrated MSNA signals were digitized at 300 samples/s by an analog-to-digital board (AT-MIO 16E2, National Instrument) and stored on the hard disk of a personal computer for off-line analysis. Thirty minutes after instrumentation, supine data acquisition during spontaneous breathing was initiated and lasted for 10 minutes (REST). At the end of REST, a blood sample was withdrawn. Thereafter, an incremental 15° step-wise head-up tilt test up to 75° (TILT) was maintained for 15 minutes. At the end of TILT, a second blood sample was withdrawn.

### Data analysis

ECG, BP, respiration signals were analyzed off line. Heart period was approximated as the time distance between two consecutive R-wave peaks (RR). SAP was taken as the maximum value of the BP signal inside the considered RR. Respiration was sampled in correspondence of each R-wave peak. All measurements were carried out on a beat-to-beat basis. The mean and the total power (i.e. variance) of RR series, μ_RR_ and σ^2^_RR_ (expressed in ms and ms^2^, respectively), and of SAP series, μ_SAP_ and σ^2^_SAP_ (expressed in mmHg and mmHg^2^, respectively) were calculated. Parametric power spectral analysis was performed utilizing 250 consecutive stationary RR, SAP and respiratory measures [21], both at REST and during a 75° head-up TILT. According to the value of the central frequency, each spectral component was labeled as low frequency (LF band, from 0.04 to 0.15 Hz) or high frequency component (HF band, from 0.15 to 0.5 Hz) [22]. The absolute power of RR and SAP series in the LF band (LF_RR_ and LF_SAP_, respectively) and of RR series in the HF band (HF_RR_) were calculated as the sum of powers of RR and SAP series components, whose central frequency dropped in LF or HF band, respectively. LF_RR_ and HF_RR_ were expressed in ms^2^, while LF_SAP_ in mmHg^2^. Normalization of RR series power in LF and HF band, i.e. LF_RRnu_ and HF_RRnu_, was obtained by dividing the LF_RR_ and HF_RR_ by total variance diminished by the power of the very low frequency band (i.e. below 0.04 Hz) and then multiplying the result by 100 [22]. LF_RRnu_ was utilized as an index of the cardiac sympathetic control, while HF_RR_ and HF_RRnu_ as markers of the cardiac vagal control [23]. LF_SAP_ was taken as an index of the sympathetic modulation directed to the vessels [21]. The LF_RR_/HF_RR_ ratio (LF/HF) quantified the sympatho-vagal interaction to the sinoatrial node [21–23].

From the MSNA signal, each sympathetic burst was automatically detected when it overcame a threshold updated on a beat-to-beat basis in order to follow both baseline oscillations and changes of MSNA burst amplitude [24]. To account for the conduction time, the burst was searched in a temporal window ranging from 900 to 1,700 ms after the R-wave peak. Burst onset and offset were detected as the points where the first derivative crossed zero. We computed three different indices from the MSNA signal: i) bursts/min, which represents the numbers of bursts per minute; ii) burst/100, i.e. the number of the bursts counted in a period of 100 cardiac beats; iii) burst/HR, i.e. the number of bursts adjusted by HR.

Blood samples were collected, refrigerated, and transported appropriately for plasma epinephrine (E), norepinephrine (NE), renin, atrial natriuretic peptides (ANP), adrenocorticotropic hormone (ACTH), serum aldosterone and cortisol. Given the known complexity and multiplicity of the response to stress [25], the choice of these biomarkers was made with the purpose of probing whether stress was present in the triathletes. The samples were centrifuged at 3000 rpm for 10 min, at 4°C. The plasma top layer was placed into Eppendorf tubes (Oldenburg, Germany) and snapped frozen and stored at −80°C until analysis. High-performance liquid chromatography with electrochemical detection (Bio-Rad Laboratories, München, Germany) was used to assess plasma E and NE. DSL 25100 Active Renin immunoradiometric assay (IRMA) was used for determination of plasma renin (Beckman Coulter, UK). Plasma ANP was measured by a specific Radioimmunoassay (RIA) (Nichols Institute Diagnostic, California, USA). An electrical Chemiluminescent Immunoassay “ECLIA” was used to assess plasma ACTH as well as serum cortisol (Cobas, Roche Diagnostics GmbH, Mannheim, Germany). A radioimmunological assay was utilized for the quantitative determination of serum aldosterone (KS17CT-100, RADIM Spa, Pomezia, Italy).

### Statistical analysis

Data are expressed as mean ± standard deviation (SD). The two-tailed unpaired Student’s *t* test was used to compare the demographic and clinical variables of the two groups. χ^2^ test was used to compare the categorical variables. Two-way repeated-measures analysis of variance with Holm-Sidak test for multiple comparison, two-factor repetition was used to evaluate the differences between the two groups during REST and TILT. Differences were considered significant at values of p<0.05. All analyses were carried out on SPSS (Statistical Package for Social Sciences, Chicago, Ill), version 17.0.

## Results

Demographic characteristics of the two groups were similar (Table 1). Athletes’ training time was 27.3±4.2 hours weekly. Their performance level over the Olympic distance triathlon (i.e. 1.5 km swimming, 40 km cycling, and 10 km running) ranged between 158 and 183 minutes for men, and between 179 and 208 minutes for women. None of the triathletes showed clinical signs of OR or OT [17]. Indeed, none of them reported a drop of their performance, nor the presence of fatigue, exhaustion, reduced training volume, modification of the post-training recovery, or other stress symptoms. Echocardiographic and CPET findings are shown in Table 2. Compared to controls, triathletes were characterized by greater left atrial (LA) diameter, left ventricular (LV) end-diastolic and end-systolic volumes, as well as thicker interventricular septum and posterior wall. Although larger than controls, the athletes’ LA and LV dimensions were still within normal limits [18]. LV ejection fraction (EF) was alike normal in both groups, as well as right ventricular indices and inferior vena cava dimensions and collapsibility. As expected, the maximal oxygen consumption at peak of the CPET was higher in the triathletes, consistent with a high level of physical conditioning [19].

**Table 1:** Demographic features of controls and triathletes.

	Controls	Triathletes	p
Gender (m/f)	8/3	7/4	0.99
Age (years)	25±2	24±4	0.333
BMI (kg/m^2^)	17.6±8.6	19.6±6.5	0.6
BSA (m^2^)	1.84±0.2	1.86±0.19	0.8

BMI indicates body mass index, BSA body surface area. Values are expressed as mean±standard deviation.

**Table 2:** Echocardiographic and CPET features of controls and triathletes.

	Controls	Triathletes	p
LAD (mm)	31.4±2.1	35±1.4	**0.001**
IVS (mm)	8±1.2	11±1.2	**0.005**
PW (mm)	8.2±0.9	10±0.5	**0.001**
LVEDV (ml/m^2^)	60±6	68.4±7.5	**0.001**
LVESV (ml/m^2^)	33.8±4.1	36.7±2.8	**0.004**
LVEF (%)	58±0.5	57±2.4	0.3
RVEDA (cm^2^)	17.2±2.6	17.5±2.2	0.7
RVESA (cm^2^)	8.7±1.4	8.5±1.8	0.7
Peak VO_2_ (ml/kg/min)	39.5±5.0	53.2±3.8	**0.003**

LAD indicates left atrial diameter, IVS diastolic interventricular septum thickness, PW diastolic posterior wall thickness, LVEDV left ventricular end-diastolic volume, LVESV left ventricular end-systolic volume, LVEF left ventricular ejection fraction., RVEDA right ventricular end-diastolic area, RVESA right ventricular end-systolic area. CPET cardiopulmonary exercise test, VO_2_ maximal oxygen consumption at peak of the CPET. Values are expressed as mean standard deviation. p<0.05 was considered as significant.

All the subjects enrolled in the study well tolerated and completed the protocol.

The spectral indices of RR and SAP variability obtained in controls and triathletes during REST and TILT are shown in Table 3. At REST, athletes were characterized by higher values of RR, σ^2^_RR_, LF_RR_ and LF_RRnu_, LF/HF, LF_SAP_, and lower values of HF_RRnu_ compared to controls. Both groups showed a physiological response to the orthostatic challenge. However, during TILT, triathletes showed greater sympathetic activation and cardiac vagal reduction compared to controls. Indeed, during orthostasis, LF_RRnu_, LF/HF were greater and HF_RRnu_ lower than in controls. Also in TILT, RR was higher in triathletes. The difference of all indices between REST and TILT was however similar in the two groups.

**Table 3:** Indices of cardiovascular autonomic control in controls and triathletes at rest and during 75° head-up tilt.

	REST		TILT	
	Controls	Triathletes	Controls	Triathletes
RR, ms	935±108	1065±68 #	652±68 *	801±157 *#
σ^2^_RR_, ms^2^	2593±1368	6371±4790 #	1580±1336	4665±5470 #
LF_RR_, ms^2^	890±557	2575±2098 #	929±754	2059±2023
LF_RRnu_	45±11	58±7 #	76±5 *	87±7 *#
HF_RR_, ms^2^	873±674	1709±1258	259±278	401±575 *
HF_RRnu_	44±8	33±8 #	19±6 *	11±6 *#
LF/HF	1.08±0.38	1.84±0.49 #	4±1 *	10±5 *#
SAP, mmHg	120±11	115±11	119±9	119±16
σ^2^_SAP_, mmHg^2^	6±5	12±7	19±14 *	18±8
LF_SAP_, mmHg^2^	2±1	4±3 #	4±3 *	10±5 *

REST indicates resting condition, TILT 75° head-up tilt, RR R-R interval, σ^2^_RR_ variance of RR interval, LF low frequency component, HF high frequency component, nu normalized unit, LF/HF low frequency/high frequency ratio, SAP systolic arterial pressure, σ^2^_SAP_ variance of SAP. Values are expressed as mean ± standard deviation.

* p<0.5 REST vs TILT

# p<0.05 controls vs triathletes.

**Fig 1:**
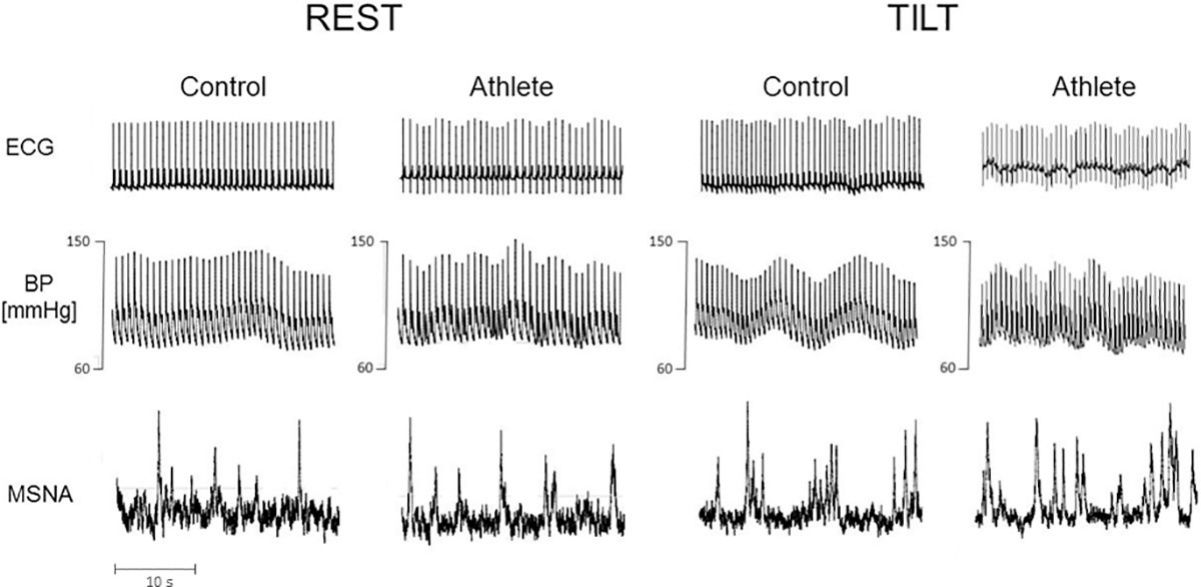
Original traces of the recorded variables in a control subject and in an athlete while supine (REST) and during head-up tilt test at 75° (TILT). Recording of 30 seconds of continuous electrocardiogram (ECG), non-invasive blood pressure (BP), and muscle sympathetic nerve activity (MSNA): despite a slower heart rate, MSNA depicts a greater sympathetic activity in the triathlete.

**Fig 2:**
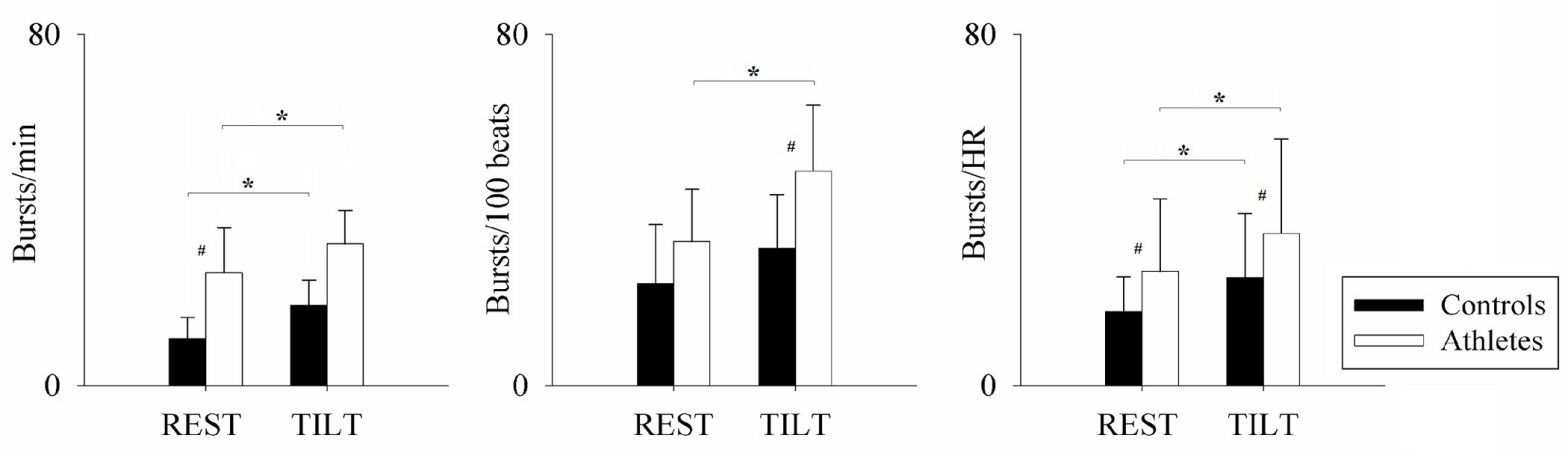
Muscle sympathetic nerve activity while supine (REST) and during head-up tilt test at 75° (TILT). Triathletes (white bars) are characterized by greater sympathetic discharge activity compared to controls (black bars), also regardless of heart rate. * p<0.05 REST vs TILT; # p< 0.05 controls vs triathletes.

Fig 1 shows representative examples of ECG, BP and MSNA recordings at REST and during TILT in a control and an athlete. The sympathetic discharge activity to the vessels assessed by MSNA burst number did increase in response to the orthostatic challenge compared to REST in both individuals. However, MSNA burst number was greater in the triathlete compared to the control subject, both at REST and during TILT. In contrast, the increase of HR during tilt was lower in the athlete. Fig 2 shows the MSNA results in the two groups, also addressing the potential confounding role of the different values of HR, given the strict link with MSNA burst occurrence [26]. Sympathetic vasomotor control was assessed by the individual values of MSNA quantified as burst rate, but also by HR-independent sympathetic activity indices, i.e. the burst/100 beats value and the burst/HR value. MSNA remained greater in athletes than in controls also when corrected for HR.

Finally, the stress mediators’ values at REST and during TILT are summarized in Table 4. At REST, E, NE, renin, aldosterone, and ANP levels were similar in the two groups. Compared to REST, E and NE physiologically increased at the end of the TILT in both groups. Otherwise, ACTH and cortisol were higher in triathletes than in controls, both during clinostatism and passive orthostatism. Cortisolemia further increased from REST to TILT only in the athletes.

**Table 4:** Stress mediators in controls and triathletes while supine and during head-up tilt test at 75°.

	REST		TILT	
	Controls	Triathletes	Controls	Triathletes
E, pg/ml	40±23	45±27	103±67*	95±46*
NE, pg/ml	293±129	353±159	625±107*	617±175*
Renin, pg/ml	9±7	10±7	8±5	9±4
Aldosterone, pg/ml	105±53	144±101	72±43	127±71
ACTH, pg/ml	15±4	73±41#	18±7	56±43#
Cortisol, ng/ml	91±42	156±92 #	86±32	180±86*#
ANP, pg/ml	69±36	72±44	94±30	95±62

REST indicates resting condition, TILT 75° head-up tilt, E plasma epinephrine, NE plasma norepinephrine, ACTH plasma adrenocorticotropic hormone, ANP plasma atrial natriuretic peptide.

* p<0.5 REST vs TILT

# p<0.05 controls vs triathletes.

## Discussion

The amateur triathletes of the present study were characterized by greater overall cardiovascular sympathetic modulation together with lower cardiac vagal activity, both at REST and during TILT, as suggested by greater LF_RRnu_, LF/HF ratio, LF_SAP_, higher burst rate of the MSNA, and lower HF_RR_ compared to controls. Accordingly, ACTH and cortisol were higher in the triathletes. Of notice, these changes were observed 72 hours after the last bout of physical exercise, and in absence of clinical OR/OT. Finally, we observed larger, although still within normal limits, left heart dimensions in the triathletes.

Previous studies have demonstrated that exercise training leads to an array of possible cardiovascular autonomic adjustments. Indices of elevated sympathetic modulation after the end of a marathon competition have been correlated to a faster arrival time [14, 27], suggesting that a transient cardiovascular sympathetic activation associated with acute intense physical activity might determine a better performance. On the other hand, regular training at intermediate load leads to chronic bradycardia and vagal predominance [28].

In the current study, triathletes were characterized by an enhanced MSNA both at REST and during TILT (Figs 1 and 2), together with higher LF_SAP_ suggestive of a sustained increased sympathetic activity to the vessels compared to controls (Table 3). This finding is further supported by the observation of higher levels of measured stress mediators (Table 4). Furthermore, the greater LF_RR_ and LF/HF ratio, and lower HF_RR_ are suggestive of enhanced sympathetic and decreased vagal modulation to the sinoatrial node (Table 3), although the physiological response to the orthostatic challenge is well preserved, not different from controls. Notably, the heavy dynamic exercise that characterizes triathlon daily training sessions may produce a cardiovascular sympathetic excitation, which outlasts the end of exercise, as already reported in other sports [10, 13]. Therefore, a resting bradycardia, associated as expected with long-term physical training, seemed to coexist with the enhanced cardiovascular sympathetic excitation as an after-effect of the intense dynamic exercise regularly performed. The latter may result in a persistent predominance of LF_RRnu_ and higher LF/HF ratio. In addition, these apparently inconsistent findings suggest that the neural control of a cardiovascular parameter, such as the RR interval, may differ from the autonomic control of its spontaneous variability [29].

In triathlon, the successful athlete must have highly developed oxygen transport and utilization systems [30]. This is accomplished by intense (2–3 hours per session) and regular (5–6 days/week) training sessions that are likely to induce a chronic sustained sympathetic activation. A greater sympathetic modulation to the heart and vessels was also observed in triathletes compared to controls during TILT, a stimulus known to enhance the overall cardiovascular sympathetic control [20, 22, 23]. This highlights the triathletes’ ability to enhance the cardiovascular sympathetic drive to the greatest levels, a condition that may favor the achievement of the best athletic performance during competitions [14, 27]. However, it must be pointed out that an exceeding sympathetic activity, when sustained, has been associated with an increased risk for cardiovascular events [13, 22, 31]. Therefore, in the amateur triathletes of the present study, the finding of greater indices of cardiovascular sympathetic activation, and lower HF_RRnu_, indicating a reduced vagal activity to the heart, even 72 hours after the last training session, raises the possibility of the presence of an unfavorable autonomic profile, potentially acting as a cardiovascular risk factor over time. This hypothesis is further corroborated by the concomitant greater plasma concentrations of ACTH and cortisol. In response to stress, the activation of the hypothalamic-pituitary-adrenal axis results in ACTH and cortisol secretion [32]. However, excessive and chronic hypercortisolemia is known to be associated with a number of untoward effects, such as an enhanced oxidative-stress and hyperlipidemia [33], impaired vascular functioning and increased platelet aggregation [34] in rats, hypertension [35] and enhanced fasting glucose levels [36] in humans. These mechanisms are thought to explain the association between stress and increased prevalence of cardiovascular disease [37]. Two recent prospective studies showed that elevated levels of cortisol predict cardiovascular death amongst elderly people with [38] and without pre-existing cardiovascular disease [39].

With regard to the larger, although still normal left heart dimensions found in these young athletes compared to controls, we might ascribe it to either the presence of the sustained high sympathetic stimulation to the heart and vessels [9, 10], or the direct hemodynamic effects induced by a regular training [40, 41], or both. However, their right chambers’ sizes, LV systolic function, and ANP levels did not differ from controls, unlike previous works that demonstrated both left and right chamber abnormalities, the presence of inflammation, fibrosis, cavity dilation, diastolic dysfunction and eccentric hypertrophy due to long-term repetitive and vigorous exercise sessions [9, 40, 42]. The young age of the participants in the present study might justify this discrepancy. Remarkably, the overall changes might ultimately lead to ‘adverse cardiac remodeling’ and promote arrhythmias in predisposing settings, such as ischemic heart disease or latent cardiomyopathies.

In the current study, we focused on triathlon, a sport that is gaining increasing popularity for practice at any age. Although in a numerically limited population, we found that triathletes are characterized by signs of chronic cardiovascular sympathetic overactivity, identified by spectral analysis of cardiovascular oscillations, muscle sympathetic nerve activity, and stress biomarkers. We hypothesize that the intense and regular training sessions are likely to induce a chronic sympathetic activation. Although such an enhanced cardiovascular sympathetic drive could have a finalistic aim, i.e. the achievement of the best athletic performance, it is compelling to consider that the induced cardiovascular sympatho-vagal modifications might also represent a potential risk factor for future cardiovascular events, such as atrial fibrillation [7], coronary artery disease [3, 43], myocardial fibrosis [3, 9] and sudden death [3–5, 11]. Indeed, a sustained sympathetic overactivity has been described in a number of pathological conditions, such as hypertension, ischemic cardiomyopathy, heart failure, diabetes mellitus, and linked to exceeding mortality and morbidity [12, 22, 44, 45].

It is worth to underline that a chronic sympathetic predominance could also be the prelude of an impending OT [46]. The triathletes tend to force up to their own perceived limit of endurance, both during training and races. The evaluation of their autonomic profile might represent a tool to unmask OR or OT, thus avoiding unfavorable effects.

Although only *ad hoc* perspective and large observational studies can address the causal relationship between exercise-induced chronic sympathetic overactivity and possible increased mortality or morbidity, the findings of the present study may carry important clinical implications highlighting the potential risk of a persistent high cardiovascular sympathetic drive induced by exercise volumes beyond the “optimal dose”, that is a current, widely debated topic [2, 3].

## Supporting information

S1 File Original data set. Original data set of the enrolled population (11 amateur triathletes and 11 age-matched healthy controls).
(XLSX)
